# Perinatal outcomes in women with class IV obesity compared to women in the normal or overweight body mass index categories: A population‐based cohort study in Qatar

**DOI:** 10.1002/osp4.698

**Published:** 2023-11-20

**Authors:** Fathima Minisha, Najat Khenyab, Salwa Abu Yaqoub, Sawsan Al Obaidly, Mai AlQubaisi, Husam Salama, Tawa Olukade, Abdul Rouf Pallivalappil, Nader Al Dewik, Hilal Al Rifai, Thomas Farrell

**Affiliations:** ^1^ Department of Obstetrics and Gynecology Women's Wellness and Research Centre Hamad Medical Corporation Doha Qatar; ^2^ Department of Pediatrics and Neonatology Women's Wellness and Research Centre Hamad Medical Corporation Doha Qatar; ^3^ Department of Pediatrics Hamad Medical Corporation Doha Qatar; ^4^ Department of Research Women's Wellness and Research Centre Hamad Medical Corporation Doha Qatar; ^5^ Chief Executive Officer Women's Wellness and Research Centre Hamad Medical Corporation Doha Qatar

**Keywords:** adverse perinatal outcomes, cesarean delivery, class IV obesity in pregnancy

## Abstract

**Background:**

The prevalence of childhood and adult obesity is rising exponentially worldwide. Class IV obesity (body mass index, BMI ≥50 kg/m^2^) is associated with a higher risk of adverse perinatal outcomes. This study compared these outcomes between women with class IV obesity and women in the normal or overweight categories during pregnancy.

**Methods:**

A retrospective cohort study was performed in Qatar, including women having singleton live births beyond 24 weeks of gestation, classified into two class IV obesity and normal/overweight (BMI between 18.5 and 30.0 kg/m^2^). The outcome measures included the mode of delivery, development of gestational diabetes and hypertension, fetal macrosomia, small for date baby, preterm birth and neonatal morbidity. Adjusted odds ratios (aOR) with 95% confidence intervals (95% CI) were determined using multivariable logistic regression models.

**Results:**

A total of 247 women with class IV obesity were compared with 6797 normal/overweight women. Adjusted analysis showed that women with class IV obesity had 3.2 times higher odds of cesarean delivery (aOR: 3.19, CI: 2.26–4.50), 3.4 times higher odds of gestational diabetes (aOR: 3.39, CI: 2.55–4.50), 4.2 times higher odds of gestational hypertension (aOR: 4.18, CI: 2.45–7.13) and neonatal morbidity (aOR: 4.27, CI: 3.01–6.05), and 6.5 times higher odds of macrosomia (aOR 6.48, CI 4.22–9.99).

**Conclusions:**

Class IV obesity is associated with more adverse perinatal outcomes compared with the normal or overweight BMI categories. The study results emphasized the need for specialized antenatal obesity clinics to address the associated risks and reduce complications.

## Graphical abstract

This is a registry‐based cohort study exploring the impact of obesity on pregnancy outcomes. The results showed that women suffering from Class IV obesity in Qatar are at a higher risk of developing gestational diabetes, gestational hypertension, cesarean delivery, and neonatal morbidity during their pregnancies compared to women in the normal or overweight categories.

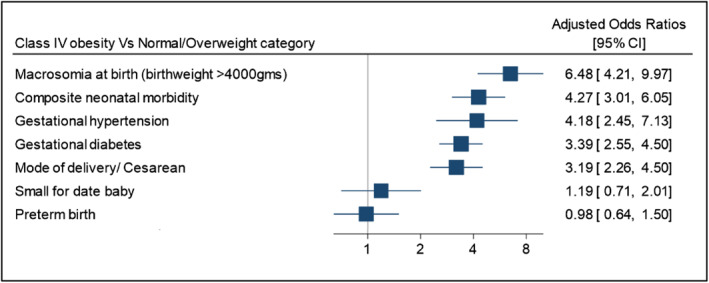

## 1 INTRODUCTION

According to the World Health Organization (WHO), in 2016, nearly 13% of the global population (over 650 million) was diagnosed with obesity, which is expected to rise to more than a billion people by 2030.^1^ The rise in prevalence over the years has been exponential and nearly 1.4 times higher in females than in males. In the Eastern Mediterranean WHO region, countries of the Middle East lead in obesity; 43% of women in Qatar were affected by obesity in 2016, second only to 46% in Kuwait. Extreme obesity has been further classified into Class IV obesity or superobesity (body mass index, BMI ≥50 kg/m^2^) due to the increasing numbers and associated health consequences.^2^

Nearly 40% of pregnant women in Qatar have obesity, with 5% in the extreme category.^3^ It is well known that pregnant women with obesity are at an increased risk of maternal and neonatal mortality and morbidity, as evidenced in the Green Top guidelines for obesity in pregnancy,^4^, ^5^, ^6^ leading to increased cost of care, decreased maternal quality of life and increased childhood disorders in the babies such as childhood obesity, diabetes, cardiovascular disorders, cognitive impairment and autism.^7^ However, most of these associations have been studied more in the broad obesity category of women with BMI ≥30 kg/m^2^ and less specifically in the higher morbidity obesity groups.

Previous studies conducted in the pregnant population of North America and Europe hint that the risks of complications increase as the maternal BMI class increases. Studies analyzing class IV obesity in pregnancy from these regions report an increased risk of maternal and neonatal outcomes^8^, ^9^, ^10^; however, it is difficult to extrapolate these results to the diverse multinational and multiethnic population of the Middle East, specifically Qatar. This is because of the differences in the clinical practice in the area and the heterogeneity within the population due to multiple nationalities and ethnicities. These previous studies either have a smaller exposed group, do not compare to women not having obesity, or do not adjust for pre‐existing comorbidities. Therefore, further research addressing these gaps and from this specific geographical area is required.

The pregnancy outcomes in the broad obesity category (BMI≥30 kg/m^2^) have been studied previously in Qatar,^11^ but none from the country or the geographical area focus on the high‐risk class IV obesity group. The increased number of pregnant women diagnosed with class IV obesity makes it essential to have focused studies quantifying the increased risk compared to those without obesity. This population‐based retrospective cohort study aimed to evaluate the various pregnancy and neonatal outcomes in women with class IV obesity compared with women in the normal/overweight BMI categories.

## 2 METHODS

### 2.1 Study design and setting

A population‐based retrospective cohort study including women giving birth in the largest tertiary maternity hospital in Qatar, averaging nearly 18,000 deliveries per year, was conducted between January 2017 and December 2020. The study was approved by the Medical Research Center, Hamad Medical Corporation (MRC‐01‐22‐028) and was exempt from informed consent since only existing data extracted after medical chart review was used.

### 2.2 Participants

The main exposure group (women with class IV obesity) was selected from all deliveries in the hospital between 2017 and 2020 to mothers with an antenatal BMI ≥50 kg/m^2^. Only singleton pregnancies with antenatally recorded BMI, gestational age ≥24 completed weeks and resulting in a live birth were included in the study. The comparison group was selected from the PEARL‐Peristat 2017 registry (Perinatal Neonatal Outcomes Research Study in the Arabian Gulf), generated using routinely collected hospital data about perinatal outcomes of all deliveries in the hospital. Women with antenatally recorded BMI between ≥18.5 and < 30 kg/m^2^ (normal/overweight BMI categories‐NO group) were included with the same exclusion criteria.

### 2.3 Data source and variables

The data for the group with class IV obesity was extracted from Cerner Millennium® patient electronic health records using the hospital numbers of women satisfying the inclusion criteria obtained from the hospital maternity records. For the NO group, all the variables were extracted directly from the PEARL 2017 dataset. The primary exposure variable was the first recorded BMI during the pregnancy, documented as a continuous variable in kg/m^2^ and then categorized based on the definitions mentioned above.

#### 2.3.1 Maternal outcomes

The following variables were studied. The mode of delivery (MOD) had two categories: vaginal delivery, VD (including spontaneous and instrumental) being the baseline group versus caesarean delivery‐CD (including elective and emergency CD for any indication). Gestational diabetes mellitus (GDM) was defined as an abnormal 75 gm glucose tolerance test in women not previously diabetic, anytime between 16 and 32 weeks of gestation, depending on the patient's risk factors.^3^ As per the hospital guidelines, all women were offered a glucose tolerance test as early as 16 weeks if they were at a high risk of acquiring GDM (e.g., with risk factors like advanced maternal age, high BMI, previous history of GDM, strong family history etc.) or even as late as 32 weeks in women with low‐risk pregnancies as evidenced by Bashir et al.^3^ Gestational hypertension (GHT) was defined in this study as the occurrence of high blood pressure after 20 weeks of gestation^12^ including preeclampsia, superimposed on chronic hypertension or eclampsia. Preeclampsia was defined as high blood pressure associated with proteinuria, and the severe form can involve the maternal hepatic, renal, pulmonary and cerebral systems.^13^

#### 2.3.2 Neonatal outcomes

The neonatal outcomes included small for date baby (SFD) defined as a fetus with the estimated weight less than 10th percentile for gestational age in the last third trimester scan.^14^ Macrosomia was defined as a birthweight ≥4000 g measured immediately after birth.^15^ Preterm birth (PTB) was defined as gestational age (GA) at birth less than 37 completed weeks.^16^ A composite neonatal morbidity (CNM) was used to represent the presence of any one of: APGAR scores less than seven in 1 min of life, admission to neonatal intensive care unit (NICU) after birth, respiratory distress syndrome (RDS) as diagnosed by the attending neonatologist, suspected or proven sepsis, prematurity (gestational age at delivery less than 32 weeks), and neonatal death (death within 1 week of life).

Data regarding potential confounders for the association between the exposure and outcomes were also collected. The maternal age was categorized using the median as the cut‐off. Parity was defined as any prior birth after 24 completed weeks‐divided into three categories: nulliparous, multiparous‐1–3 prior births, and grand multiparous‐four or more prior births.^17^ Other variables included pre‐existing medical comorbidities such as diabetes, chronic hypertension, thyroid disorders, asthma, cardiovascular and renal disorders, history of bariatric surgery and history of assisted reproduction.

Age and parity were considered a priori confounders for all maternal outcomes. Other confounders were identified based on past literature and using directed acyclic graphs (DAG) as shown in the Figure S1. Variables that fell in the causal pathway between BMI and the outcomes were excluded from the models in order to obtain the direct causal effect of the exposure on the outcomes (for example‐assisted reproduction was on the pathway between BMI and gestational hypertension or preterm birth and hence not included in the models).

### 2.4 Sample size

The sample size was estimated for three main outcomes–MOD, GDM and GHT. The WHO estimates showed nearly 20% baseline CD risk in the women delivering in Qatar.^18^ This risk was nearly 30% in the country in women with BMI ≥40 kg/m^2^ ^11^; this was estimated to increase to at least 35% in the group with class IV obesity. The baseline risk of GDM and GHT was 21.5% and 5%, respectively.^3^, ^19^ Based on previous publications, a risk of at least 45% GDM and 13% GHT in the group with class IV obesity was expected.^11^ The minimum sample size required per group to detect these differences in proportions with a power of 80% and a false positive rate of 5% based on a 2‐tailed Chi‐square test and accounting for 10% missing data, was atleast 225 women.

### 2.5 Data management and analysis

Data collected as continuous variables (age, BMI, parity, GA at birth, number of previous CDs, and birth weight) were categorized according to the predefined criteria and coded appropriately (all baseline groups coded 0). Women aged less than 15 years or more than 49 years were excluded. Variables with counts less than five were either recategorized or not reported to maintain patient confidentiality.

Continuous variables were reported as mean ± standard deviation (SD) or median ± interquartile range (IQR) based on the distribution of the variables (assessed using histograms and/or Shapiro‐Wilk test) and compared using Student's *t*‐test and Wilcoxon rank‐sum test as appropriate. Categorical variables were reported as frequency and percentage and compared using Chi‐square or Fisher's exact test as appropriate.

Crude odds ratios (OR) and 95% confidence intervals (CIs) were obtained using logistic regression models for each outcome. Adjusted ORs (aORs) for each association were obtained after adjusting for confounders (avoiding variables on the causal pathway and assessing for multicollinearity). Date sparsity was handled by avoiding variables having <10 outcomes. The number of parameters in the models was restricted to one‐tenth of the total outcome counts.

The null hypothesis stated that no difference existed between the groups beyond chance. A *p*‐value less than 0.05 was evidence against the null hypothesis, concluding that a difference existed beyond mere chance. All analyses were done in Stata statistical software, Release 16.^20^

## 3 RESULTS

### 3.1 Characteristics of the study population

A total of 7044 women satisfied the inclusion criteria–247 women with class IV obesity and 6797 women in the normal or overweight categories. Over the study period, nearly 62 women with class IV obesity had singleton viable live births per year‐an average of 3.4 per 1000 live births.

The median BMI of the group with class IV obesity was 51.9 kg/m^2^ (IQR 50.7–54.3), with the highest BMI being 68.7 kg/m^2^. The mean age of these women was 32.9 years (±SD 5.2) compared to 28.3 ± 5.4 years in the NO group (*t*‐test *p*‐value <0.001). A higher proportion in this group were older (73% vs. 40%), Qatari (36% vs. 26%) and grand multiparous women (32% vs. 10%) as shown in Table 1.

**TABLE 1:** Maternal demographics between the exposure groups.

Maternal demographics	Women with class IV obesity (BMI ≥50)		Women with normal/overweight BMI (≥18.5 to <30)		*p*‐value
	*N* = 247		*N* = 6797		
	*n*	%*N*	*n*	%*N*	
Age categories					
<30 years	67	27.1	4084	60.1	<0.001*
≥30 years	180	72.9	2713	39.9	
Nationality					
Qatari	90	36.4	1793	26.4	<0.001*
Non‐Qatari	157	63.6	5004	73.6	
Parity					
0: Nulliparous	36	14.6	2254	33.1	<0.001*
1–3: Multiparous	133	53.8	3886	57.2	
≥4: Grand multiparous	78	31.6	657	9.7	
Previous CD					
0	143	57.9	5692	83.7	<0.001*
≥1 CD	104	42.1	1105	16.3	
Assisted conception	7	2.8	100	1.5	0.103
History of bariatric surgery	15	6.1	92	1.4	<0.001*
Chronic hypertension	17	6.9	39	0.6	<0.001*
Preexisting diabetes	33	13.4	93	1.4	<0.001*
Thyroid disorders	49	19.8	492	7.2	<0.001*
Asthma	15	6.1	119	1.8	<0.001*

*Note*: Comparison using Chi‐square/Fisher's exact.

Abbreviations: CD, cesarean delivery; GA, gestational age.

*p < 0.05 strong evidence against null hypothesis of no difference.

**FIGURE 1:**
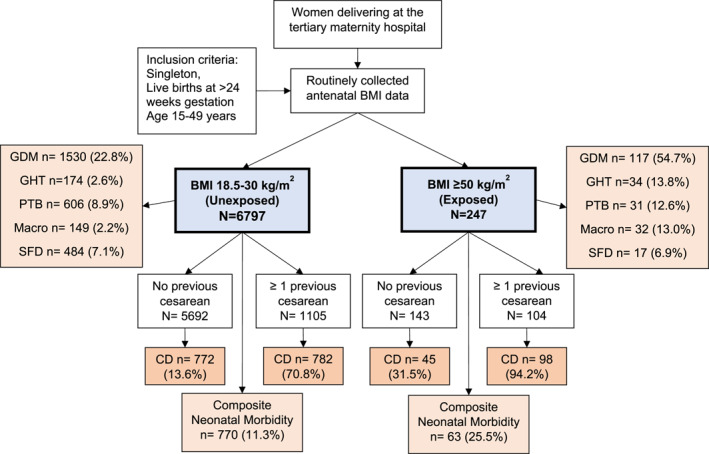
Flow chart showing exposure groups and outcomes; CD, cesarean delivery; GDM, gestational diabetes; GHT, gestational hypertension; PTB, preterm birth Macro, Macrosomia at birth; SFD, small for date baby; Percentage, *n*/*N* *100.

In addition, 42% had a previous CD compared to only 16% in the NO group (Figure 1). The women with class IV obesity had significantly higher proportions of chronic hypertension, pre‐existing diabetes, thyroid disorders and asthma. Additionally, 6% had a history of bariatric surgery compared to 1% in the NO group. Pre‐existing cardiovascular disease and renal disorders are not reported due to low numbers.

### 3.2 Maternal outcomes

Table 2 shows the pregnancy outcomes in the exposure groups, with the crude and adjusted ORs. A total of 1697 women in the study delivered by caesarean delivery (only 44.5% of whom delivered by a planned elective caesarean). The remaining 5347 women delivered vaginally, 495 of whom needed instrumental delivery (7.7% of vaginal births in women with class IV obesity and 9.3% in the NO group). The CD risk in the group with class IV obesity was 58% (vs. 23%), with 88% of them completing 37 weeks of gestation. In these women with more than one previous CD, 94% delivered by a repeat caesarean, compared to 71% in the NO group. The group with class IV obesity had a 4.6 times higher odds of delivery by CD. After adjusting for confounders such as age, parity, pre‐existing comorbidities, and previous CDs, this group still had a 3.2‐fold higher odds of delivery by CD (aOR 3.19, 95% CI 2.26–4.50; *p* < 0.001).

**TABLE 2:** Crude and adjusted analysis of the outcomes among the exposure groups.

Perinatal outcomes	Women with class IV obesity		Women with normal and overweight BMI		Crude OR (95% CI)	Adjusted OR (95% CI)	Wald *p*‐value
	*N* = 247		*N* = 6797				
	*n*	%*N*	*n*	%*N*			
Mode of delivery							
Vaginal	104	42.1	5243	77.1	1	1	<0.001*
Cesarean	143	57.9	1554	22.9	4.64 (3.58–6.01)	3.19 (2.26–4.50)	
Gestational diabetes	117	54.7	1530	22.8	4.08 (3.10–5.37)	3.39 (2.55–4.50)	<0.001*
Gestational hypertension	34	13.8	174	2.6	6.08 (4.11–8.99)	4.18 (2.45–7.13)	<0.001*
Preterm birth	31	12.6	606	8.9	1.41 (0.96–2.09)	0.98 (0.64–1.50)	0.942
Small for date baby	17	6.9	484	7.1	0.96 (0.58–1.59)	1.19 (0.71–2.02)	0.511
Macrosomia at birth	32	13.0	149	2.2	6.64 (4.43–9.96)	6.48 (4.22–9.99)	<0.001*
Composite neonatal morbidity	63	25.5	770	11.3	2.68 (1.99–3.60)	4.27 (3.01–6.05)	<0.001*

*Note*: Gestational diabetes proportions excluded women with pre‐existing diabetes. Mode of delivery adjusted for maternal age, parity, previous cesarean section, pre‐existing diabetes, hypertension, asthma, thyroid disease and history of bariatric surgery. Gestational diabetes adjusted for maternal age, nationality, parity, chronic hypertension, thyroid disease and bariatric surgery. Gestational hypertension adjusted for maternal age, nationality, parity, pre‐existing diabetes, thyroid disease and bariatric surgery. Preterm birth and small for date baby adjusted for maternal age, parity, previous cesarean section, pre‐existing diabetes and hypertension, and bariatric surgery. Macrosomia adjusted for maternal age, parity, and pre‐existing diabetes. Composite neonatal morbidity adjusted for age, parity, pre‐existing diabetes, chronic hypertension, and low birthweight.

Abbreviations: CI, confidence intervals; OR, odds ratio.

*p < 0.05 strong evidence against null hypothesis of no difference.

More than 54% of women with class IV obesity developed GDM, and 14% had GHT, compared to 23% and 3% in the NO group (Figure 1). After adjusting for confounders, these women had a 3.4‐fold higher odds of developing GDM (aOR 3.39, 95% CI 2.55–4.50; *p* < 0.001) and 4.2‐fold higher odds of developing GHT (aOR 4.18, 95% CI 2.45–7.13; *p* < 0.001).

### 3.3 Neonatal outcomes

The mean birthweight in the group with class IV obesity was 3325 ± 615 g (compared to 3091 ± 517 g in the NO group, *t*‐test *p*‐value <0.001), with the highest birthweight in this group being 4670 g and 13% of the newborn macrosomic (vs. 2% in NO women). They had 6.5 times higher odds of having a macrosomic baby after adjusting for confounders (aOR 95% CI, 4.2–9.9; *p* < 0.001). Both groups had similar odds of having an SFD baby or preterm birth.

**FIGURE 2:**
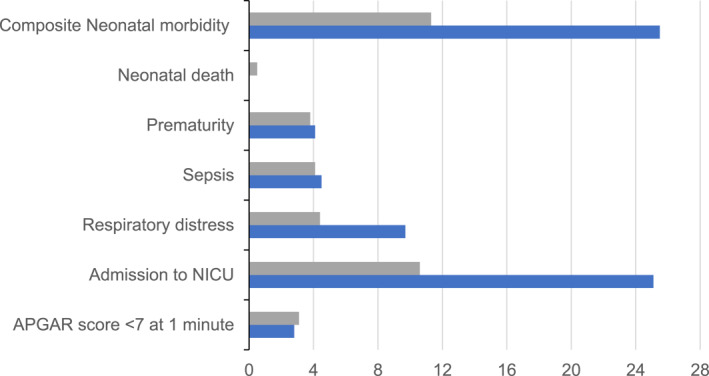
Percentages of neonatal outcome in each comparison group, Blue‐women in class IV obesity category; Gray‐women in normal or overweight BMI category.

In the class IV obesity group, 26% of the babies had at least one neonatal complication compared with 11% in the NO group. Similarly, respiratory distress and admission to the NICU were significantly more common, although low APGAR scores at birth, prematurity, and sepsis were similar (Figure 2). The adjusted analysis shows that babies born to mothers with class IV obesity had 4.3 times higher odds of having a composite neonatal morbidity (aOR 4.27, 95% CI, 3.01–6.05; *p* < 0.001).

## 4 DISCUSSION

**FIGURE 3:**
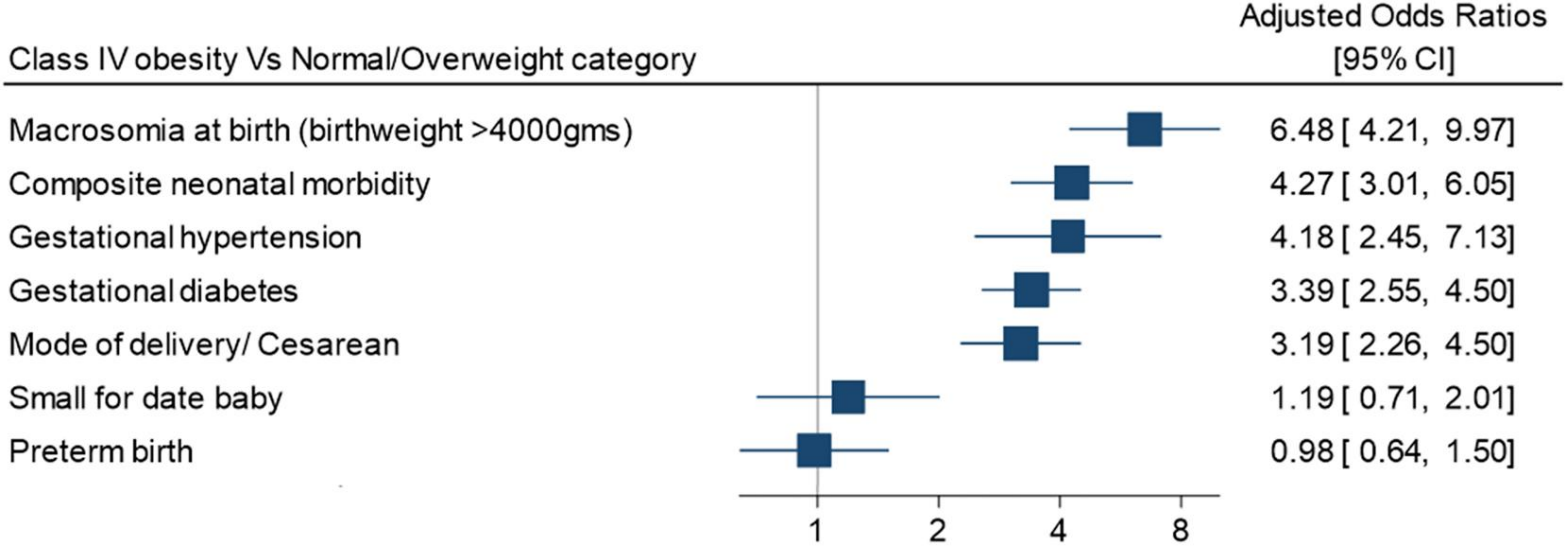
Adjusted Odds ratios (OR) and 95% confidence intervals (CI) for the association between the exposure and various outcomes; estimates obtained from multivariable logistic regression models for each outcome.

The study results provided strong evidence for an association between class IV obesity and adverse pregnancy outcomes. After adjusting for potential confounders, the odds of caesarean delivery and gestational diabetes were more than three times higher in the group with class IV obesity, more than four times higher for gestational hypertension and neonatal morbidity, and nearly 6.5 times higher for macrosomia (all associations with strong evidence against the null hypothesis; *p* < 0.05). The aORs and 95% CIs are displayed in Figure 3.

Some key differences between this study and previous literature on class IV obesity in pregnancy need to be highlighted. For example, a retrospective cohort study in the US reported a 49% CD risk in their cohort of 1185 women with class IV obesity, compared to their national CD risk of 32%.^8^ However, they excluded women with pre‐existing comorbidities, GDM and preterm births, which likely reflects the lower CD rate and lower risk of neonatal morbidity compared to this study. A 2010 UK Obstetric surveillance system study reported a 50% CD risk and 11% risk of GDM in 665 women with extreme obesity; however, adjusted results were unavailable.^21^

On the other hand, an Australian cohort study, including 370 women with class IV obesity, reported a much more comparable CD risk of 52% as they did not have a restrictive inclusion criteria.^10^ However, they reported 2.5 times higher odds of GDM in the group with class IV obesity, lower than the OR of 3.4 presented here. This difference is because their comparison group included all women with BMI <50 kg/m^2^ and therefore would have a higher incidence of GDM than the women in the normal or overweight BMI categories in this study. In addition, they reported a much higher odds of developing GHT and preeclampsia; however, they did not adjust for any pre‐existing maternal medical disorders such as chronic hypertension and pre‐existing diabetes.

A Canadian study in 2013 compared 71 women with class IV obesity to 5717 women with normal or overweight BMI (similar to the definition of comparison groups in this study).^9^ They reported a CD risk of 61% in their obesity group and 1.5 times higher odds compared to the control. In addition, they reported a GDM risk of 21% and 20% risk of GHT, contrasting with 54% and 14% risk reported here. The difference in the risk of GDM is likely explained by differences in the testing regime and diagnostic criteria used for GDM diagnosis, with the protocols in Qatar having a lower threshold for the diagnosis of GDM.

Shaukat et al. examined pre‐pregnancy BMI and maternal outcomes in Qatar, comparing nulliparous women with obesity and women in the overweight and normal BMI categories. They reported a 25% risk of CD, 41% GDM and 7.5% GHT in women with obesity (BMI ≥30); this contrasts with the risks of 58%, 54% and 14%, respectively reported here, when studying only the category with class IV obesity, providing more evidence for the dose‐response effect of BMI on adverse outcomes.^11^ Furthermore, they reported a 3% rate of macrosomia and 11% NICU admission compared to 13% and 25% reported in this study. Since the lower obesity categories have already been studied in Qatar, these categories are not being analyzed in this study. Additionally, this study was powered to study the difference between class IV obesity and the NO group; the number analyzed in this study was not enough to detect meaningful differences between class IV obesity and the other obesity groups. The possible dose‐response effect of BMI on perinatal outcomes must be corroborated further with larger studies, including all BMI categories and robust statistical analyses.

This study emphasized the higher risk of pregnancy complications associated with higher classes of maternal obesity, leading to increased short‐ and long‐term hospital costs. The results from this study can aid in pre‐natal counseling of women with high BMI in the reproductive age group as an incentive for weight reduction before conception, which will help them have healthier pregnancies.

The care should ideally begin from the adolescent age group‐counseling regarding healthy diet options and active lifestyle must begin in young girls so that they enter their reproductive years with healthy body weights. These results would also help in the counseling before assisted reproduction and help place further restrictions on the criteria for accessing these services to become pregnant.

Disseminating these results will increase the awareness among obstetricians about the magnitude of the risk and therefore be able to effectively risk‐assess pregnancies affected by maternal class IV obesity. In addition, several policies can be implemented, for example, setting up dedicated maternal obesity clinics that will provide holistic care for pregnant women with higher classes of obesity to help them achieve better outcomes. This service would also include following these women postnatally to encourage further weight reduction and a healthy BMI before starting another pregnancy.

The analysis excluded variables in the causal pathway between BMI and the outcomes. It would be interesting to explore the impact of these mediators on the associations since women with higher BMI often have difficult obstructed labor resulting in CDs, regardless of other risk factors. Other important questions that can be explored in the future would be the impact of bariatric surgery on the outcomes or the impact of controlling weight gain during pregnancy in this high‐risk BMI group.

Although there is available literature focusing on obesity and pregnancy outcomes, this study was the first to report the same in this extreme BMI group from Qatar and the Middle East. This area has a rising prevalence of adolescent obesity, which means the average BMI of women entering their reproductive age group is rising,^22^ the main culprits being the shift in lifestyle habits and urbanization at the turn of the 21st century in the middle and high‐income countries of this region. Therefore, the results of this study will help inform risk management strategies for this high‐risk group.

The women in the study were representative of the pregnant women in the country, as more than 80% of the deliveries in the country occurred at the study site. This study provided valuable real‐world insight into Qatar's heterogeneous population consisting of women from atleast 96 different countries, as it is often difficult to extrapolate results from studies conducted in more homogenous populations in other parts of the world.

The completeness of the data is another strength of this study‐all variables except one had complete data, with no missing data in the class IV obesity group. The data were collected by well‐trained personnel with the knowledge and expertise to navigate the electronic records and interpret medical documentation accurately. The study was well‐powered to detect the differences in the main outcomes with 247 women in the class IV obesity group (more than what was estimated). A priori assessment of possible confounders from previous studies and exploration of the relationships between the variables in the dataset was performed using conceptual frameworks before statistical analysis. This helped eliminate variables on the causal pathway from being included in the models.

However, some limitations need to be highlighted. The study results are limited to the women satisfying the inclusion criteria. Miscarriages (pregnancies ending before 24 completed weeks), multiple gestations and stillbirths or intrauterine fetal deaths were excluded, underestimating the incident risk of GDM and GHT in the obesity group, as they represent significant risk factors for the same. Misclassification of the exposure groups was possible due to human error in entering the BMI information in the medical charts; however, all women with class IV obesity had their BMI information cross‐checked and were found to be accurate. Misclassification of the outcome was possible with GDM‐since many women chose not to undergo the test. This is likely to be more in the NO group (as women with higher BMI are more likely to adhere to testing protocols strictly).

Residual confounding was still a problem even after adjusting for various factors. For example, the heterogeneity in the population (due to the huge mix of genetic ethnicities) was challenging to adjust for just by using nationality as a binary variable (since ethnicity is different from nationality). In addition, retrospective data collection from medical records had the drawback of incomplete documentation resulting in missed important history such as past medical illness (despite frequent hospital checks for completeness of entries by documentation specialists). These limitations must be kept in mind while interpreting the findings.

## 5 CONCLUSION

In conclusion, in this study, women with class IV obesity had poorer pregnancy and neonatal outcomes than women in the normal or overweight BMI categories, having more than thrice the odds of CD and GDM and more than four times the odds of GHT and neonatal morbidity. In light of these findings, setting up services specifically for these women might be warranted. Further prospective studies are required to study the impact of prevention policies and interventions undertaken in this high‐risk group on maternal and fetal health.

## CONFLICT OF INTEREST STATEMENT

The authors declare no conflicts of interest.

## Supporting information

Figure S1
