# Supplementary material for: Perinatal outcomes in women with class IV obesity compared to women in the normal or overweight body mass index categories: A population‐based cohort study in Qatar
Source: Obes Sci Pract. 2023 Nov 20;10(1):e698. doi: 10.1002/osp4.698 (PMC10804329; doi:10.1002/osp4.698)
Supplement: Supplementary file 1 — Figure S1 [file OSP4-10-e698-s001.docx]

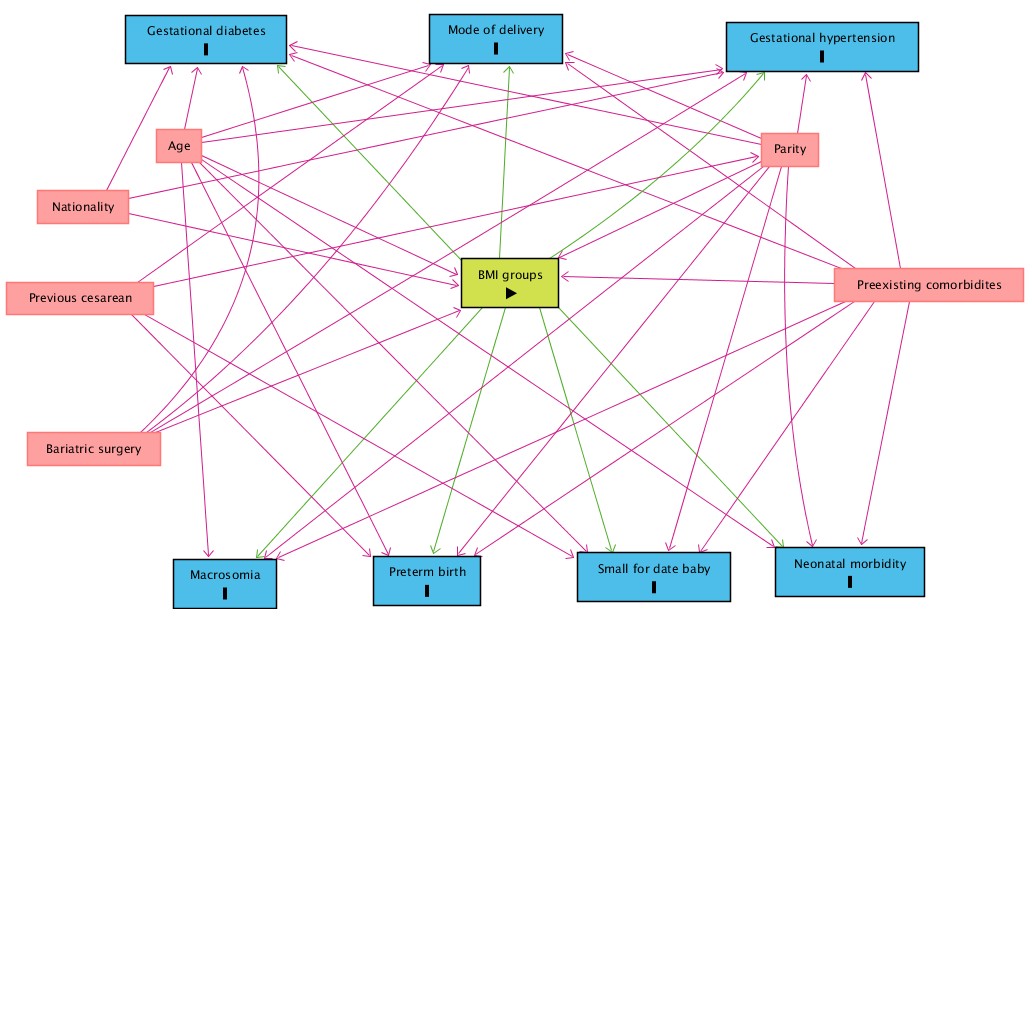

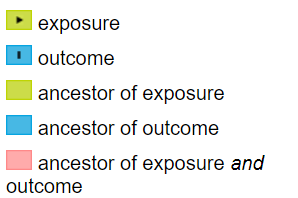

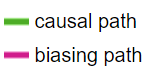


**Figure S1: Directed acyclic graphs showing main exposure (BMI groups) and the various outcomes (blue boxes with I). The variables in pink boxes represent the various confounders. (generated using www.daggity.net)**
